# Heat Capacities of α-, β-, and γ- Polymorphs of Glycine

**DOI:** 10.3390/molecules29225366

**Published:** 2024-11-14

**Authors:** Václav Pokorný, Vojtěch Štejfa, Jakub Havlín, Michal Fulem, Květoslav Růžička

**Affiliations:** 1 Department of Physical Chemistry, University of Chemistry and Technology, Prague, Technická 5, CZ-166 28 Prague, Czech Republic; pokorny@imc.cas.cz (V.P.); stejfav@vscht.cz (V.Š.); fulemm@vscht.cz (M.F.); 2 Institute of Macromolecular Chemistry, Czech Academy of Sciences, Heyrovského nám. 2, CZ-162 06 Prague, Czech Republic; 3 Central Laboratories, University of Chemistry and Technology, Prague, Technická 5, CZ-166 28 Prague, Czech Republic; havlinj@vscht.cz

**Keywords:** glycine, crystalline phase, polymorphism, heat capacity

## Abstract

As a part of our effort to establish reliable thermodynamic data for amino acids, the heat capacity and phase behavior are reported for two stable polymorphs (α and γ) of glycine (aminoacetic acid, CAS RN: 56-40-6). Prior to heat capacity measurement, thermogravimetric analysis and X-ray powder diffraction were performed to determine decomposition temperatures and initial crystal structures, respectively. The literature heat capacities obtained by adiabatic calorimetry are available in the temperature interval (7–304). The literature data were used for validating performance of our relaxation (heat-pulse) calorimeter, which was used for measurement of the heat capacity of α-glycine in the temperature interval (2–267) K. The crystal heat capacities of the α- and γ-glycine were extended towards higher temperatures using Tian–Calvet calorimetry in the temperature interval (262–358) and power compensation DSC in the temperature interval (310–449) K. As a result, reference heat capacities and thermodynamic functions for the crystalline phase from 0 K up to 450/435 K for α/γ-glycine were developed. The literature heat capacities for β-glycine over the range 0 K to 295 K were treated in the same manner in order to provide thermodynamic data for all three polymorphs existing at the atmospheric pressure.

## 1. Introduction

This work is a continuation of our project, the goal of which is to establish reliable thermodynamic data for a group of proteinogenic amino acids [1,2,3,4]. Glycine is the simplest and only achiral proteinogenic α-amino acid. Like other α-amino acids, it exists in the crystalline phase as a zwitterion. Glycine has been shown to exist in several polymorphic modifications [5,6,7], and other polymorphs have been predicted computationally [8]. Only three polymorphs (α, β, and γ) have been observed at ambient temperatures and pressures, which differ in their physical properties and biological activity. The monoclinic polymorph β is reportedly unstable [9]; at temperatures above 295 K, it gradually changes to polymorph α or γ. The monoclinic α-form has been found to have an enantiotropic relationship with the trigonal γ-form and is slightly less thermodynamically stable at low temperatures [10,11,12]; however, its transformation into the γ-form appears to be kinetically hindered.

On heating, polymorph γ changes to polymorph α at a temperature of approximately 440 K [12,13]; the reverse transformation has never been observed on cooling [14,15]. It follows that mainly the stable polymorphs α and γ are of interest for major applications, including, for example, the pharmaceutical and food industries. At ambient temperature and pressure, glycine is a white crystalline substance and is reported to decompose at/before melting; depending on the conditions used, decomposition is reported over a fairly wide temperature range of 472–565 K [16,17].

Glycine plays a crucial role in numerous biological and metabolic processes. It was also detected in outer space [18], and its role in the origin of terrestrial life is being discussed [19]. Although it is classified as a non-essential amino acid for humans, its importance goes beyond basic protein synthesis. Glycine is an essential component of key biological molecules such as creatine, glutathione, heme, purines, and porphyrins [20]. It actively participates in metabolic reactions and physiological regulation. Additionally, glycine serves as a major inhibitory neurotransmitter in the spinal cord and brainstem, contributing to neurological functions. It offers cytoprotective, anti-inflammatory, and immune-modulating benefits [21]. The therapeutic potential of glycine is well documented: it helps prevent tissue injury, promotes protein synthesis, aids in wound healing, and enhances immune functions. Glycine supplementation is also associated with the treatment of various metabolic disorders, including obesity, diabetes, cardiovascular diseases, and certain inflammatory conditions [22].

Despite the well-recognized biological and technological importance of glycine, there is a notable lack of reliable thermodynamic data for this amino acid, particularly above the ambient temperature. A high proportion of the previously published data do not explicitly indicate which polymorph has been investigated. Moreover, glycine is typically sold as a mixture of α- and γ-polymorphs, and obtaining a pure polymorph is not a straightforward task [11,15].

In this study, new experimental crystal heat capacities for α- and γ-glycine are reported. After establishing the crystal structure of both polymorphs by X-ray powder diffraction (XRPD), the decomposition temperature was determined by thermogravimetry. Heat capacities in the temperature range (262 to 358) K were determined by Tian–Calvet calorimetry and extended up to 434 K or higher by power compensation DSC. The literature low-temperature heat capacities were found for α-, β-, and γ-glycine (adiabatic calorimetry from ca. 7 K to ca. 300 K). New measurements were taken for α-glycine using thermal-relaxation calorimetry (from 2 K to 267 K) to complement previously performed performance testing of this technique [23] using different sample encapsulation methods.

The heat capacities of this work are combined with the selected literature values and fitted as a function of temperature from 0 K to 450 K (α-glycine) and up to 435 K (γ-glycine). The same procedure was applied for the available literature heat capacities of β-glycine from 6 K to 295 K. The recommended values of the standard thermodynamic functions were calculated for α-, β- and γ-glycine in the above temperature ranges.

The phase change of γ-glycine to its α-form was then studied using heat-flux differential scanning calorimetry (HF DSC) in order to establish more reliable temperature and enthalpy of this phase transition.

## 2. Results and Discussion

### 2.1. Thermogravimetric Analysis (TGA)

**Figure 1:**
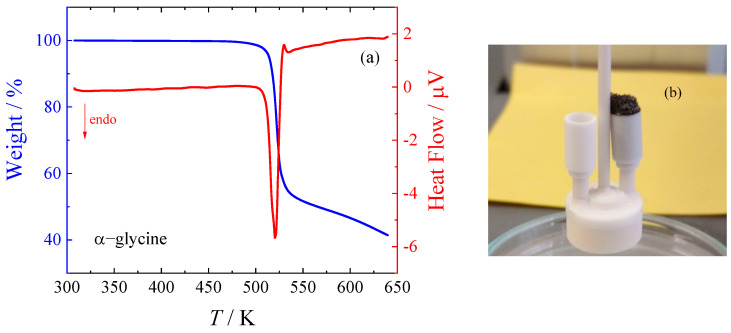
TGA analysis of α-glycine using SETARAM Setsys Evolution (SETARAM, Caluire-et-Cuire, France): (**a**) thermogram; (**b**) visual appearance after TGA.

Traditionally, amino acids are reported to melt or decompose at temperatures above 450 K [16,17]. However, the literature data exhibit a significant scatter. Moreover, a recent paper by Weiss et al. [24] suggested that all amino acids studied do not melt but rather decompose, which could damage calorimeters as a result (see Figure 1b). Weiss et al. [24] reported an evolution of CO_2_, NH_3_, and H_2_O during decomposition of glycine (unspecified polymorph) at the temperature of about 503 K to 523 K (peak onset and top, respectively).

α-Glycine was studied by TGA in this work, and the results are shown in Figure 1a. The simple one-stage decomposition was observed.

A summary of the decomposition temperatures obtained in this work as well as those reported in the literature is presented in Table 1. It should be noted that the decomposition kinetics are influenced by a number of factors (heating rate, sample size, purge gas, etc.); this is reflected by the relatively large range of reported decomposition temperatures.

Decomposition can be avoided by using fast scanning DSC, capable of scanning rates up to 20,000 K·s^−1^. Though this instrumentation is commercially available, such data are extremely scarce in case of amino acids and originate from the same collective of authors [25,26]. In case of glycine, reported temperatures of melting *T*_fus_ [25,26] are higher than those of decomposition by 10 to 95 K. Fast scanning DSC is also capable of providing the enthalpies of fusion needed, for example, for solubility modeling. Heat capacities at temperatures well below *T*_fus_ must be known for this purpose, as they are used for mass determination of tiny samples. In studies [25,26], the heat capacities published by Parks et al. [27] and Hutchens et al. [28] were used in this procedure.

**Table 1:** Decomposition/melting temperatures of glycine ^a^.

Reference	Event	*T*/K	Method	Scanning Rate, Purge Gas
SRC recommendation ^b^	decomposition	535		
Contarini and Wendlandt [29]	decomposition	535	DSC	10 K min^−1^, nitrogen
Contarini and Wendlandt [29]	decomposition	561 ^c^	TVD ^d^	10 K min^−1^, nitrogen
Contarini and Wendlandt [29]	decomposition	558 ^e^	TGA	10 K min^−1^, nitrogen
Rodriguez-Mendez et al. [30]	decomposition	502 ^f^	TGA	10 K min^−1^, air
Rodriguez-Mendez et al. [30]	decomposition	500 ^f^	DTA	10 K min^−1^, air
Rodante et al. [31]	decomposition	499 ^f^	TG-DSC	10 K min^−1^, nitrogen
Rodante et al. [31]	decomposition	529 ^c^	TG-DSC	10 K min^−1^, nitrogen
Wesolowski and Erecinska [32]	decomposition	498 ^c^	DTA	5 K min^−1^, air
Wesolowski and Erecinska [32]	decomposition	503 ^c^	DTG	5 K min^−1^, air
Srinivasan [33]	decomposition	500 ^f^	DTA	10 K min^−1^, nitrogen
Weiss et al. [24]	decomposition	523 ^c^	TGA	5 K min^−1^, argon
This work	decomposition	512	TGA	5 K min^−1^, argon
Chua et al. [25]	melting	569 ± 7	FSC ^g^	(2–10) × 10^3^ K min^−1^, nitrogen
Do et al. [26]	melting	569 ± 9	FSC ^g^	(2–10) × 10^3^ K min^−1^, nitrogen

^a^ Sources where melting/decomposition temperature is merely mentioned are not listed. Values in this table are rounded to the nearest kelvin. ^b^ “Temperature of fusion” (in fact, temperature of decomposition) recommended by the Syracuse Research Corporation and used by many authors (retrieved from CAS SciFinder [34]). ^c^ Peak maximum temperature. ^d^ TVD stands for thermovoltaic detection. ^e^ Temperature for maximum slope of TG curve. ^f^ Start of the peak (onset not reported). ^g^ FSC stands for fast scanning calorimetry.

### 2.2. Heat Capacities

The experimental heat capacities of α- and γ-glycine obtained in this work with the Tian–Calvet calorimeter SETARAM μDSC IIIa (SETARAM, Caluire-et-Cuire, France) and PerkinElmer power compensation (PC) DSC 8500 (PerkinElmer, Waltham, MA, USA) are listed in the Supplementary Materials (SM) in Tables S1 and S2, including the correction scaling factors applied for PerkinElmer DSC 8500. The results for α-glycine obtained by Quantum Design PPMS relaxation calorimeter (Quantum Design, San Diego, CA, USA) are given in Table S3. The available literature data on crystal heat capacities are summarized in Table 2.

The experimental data of this work and selected literature values (marked bold in Table 2) were fitted with Equations (2) and (3), the parameters of which are given in Table 3.

**Figure 2:**
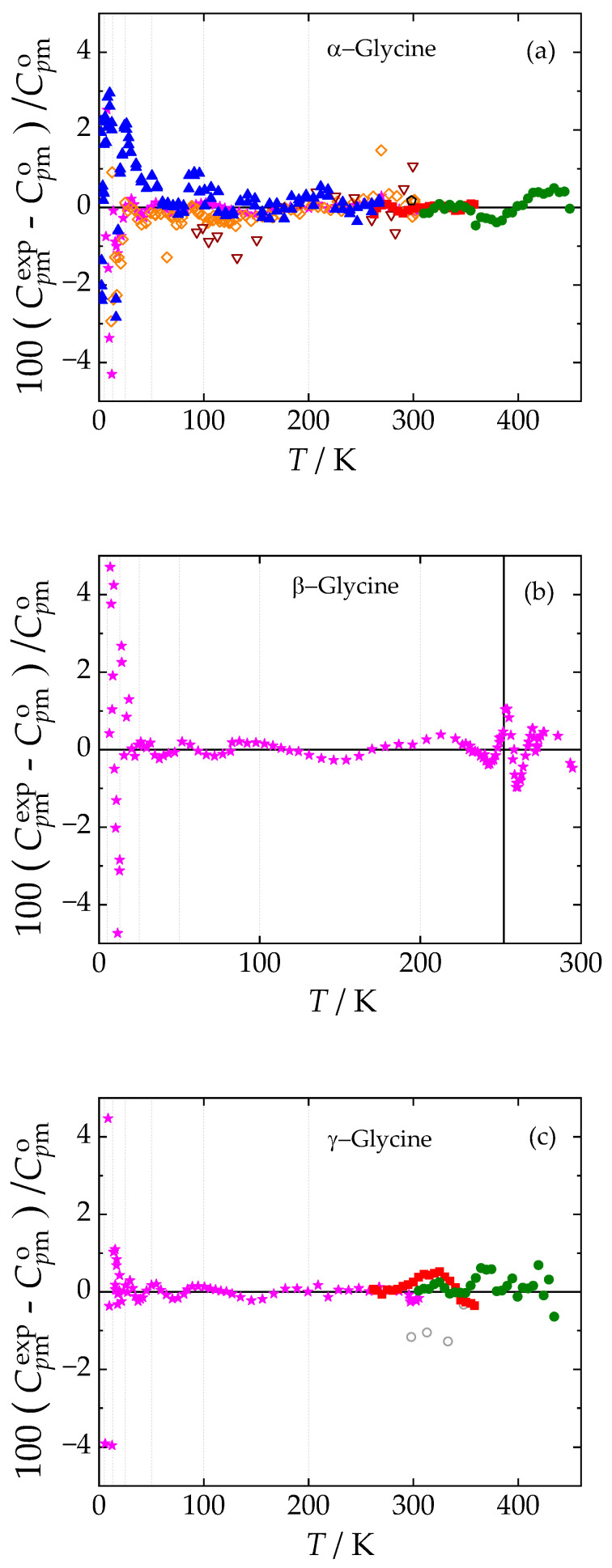
Relative deviations $100\left(C_{pm}^{\exp}-C_{pm}^{o}\right)/C_{pm}^{o}$ of individual experimental heat capacities $C_{pm}^{\exp}$ from values $C_{pm}^{o}$ calculated by means of Equations (2) and (3) with parameters from Table 3. (**a**) α-glycine, (**b**) β-glycine, and (**c**) γ-glycine. Blue 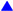, this work (relaxation calorimetry); red 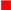, this work (Tian–Calvet calorimetry); green 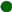, this work (power compensation DSC); brown 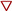, Parks et al. [27]; orange 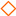, Hutchens et al. [28]; black 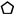, Spink and Wadsö [35]; magenta 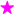, Drebushchak et al. [9,12]; gray 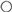, Badelin et al. [37]. Vertical lines denote the knot temperatures *T*_i_ (dotted lines) or transitions (solid lines). Data points shown as empty symbols were not included in the fit.

All the experimental as well as literature heat capacities for α-, β-, and γ-glycine are compared with the smoothed values obtained using Equations (2) and (3) in Figure 2. The deviations of the selected experimental data from the smoothed values do not exceed 1%, with the exception of lowest temperatures (where all experiments have higher uncertainty) and the vicinity of a ferroelectric–paraelectric transition of β-glycine (as discussed in [9]), where the data points were excluded from the fit. It should be noted that prior to the adiabatic measurements, which were made by a joint effort of several institutions in Novosibirsk between 2003 and 2005 [9,12], no information was given for the measured heat capacities as to which polymorph was being studied. Whether it is an α- or γ-polymorph can be inferred from the measured values, but it cannot be ruled out that it was a mixture of both polymorphs. For example, Drebushchak [12] reported that his sample of polymorph α contained 6% of polymorph γ, and the published results were corrected accordingly.

**Figure 3:**
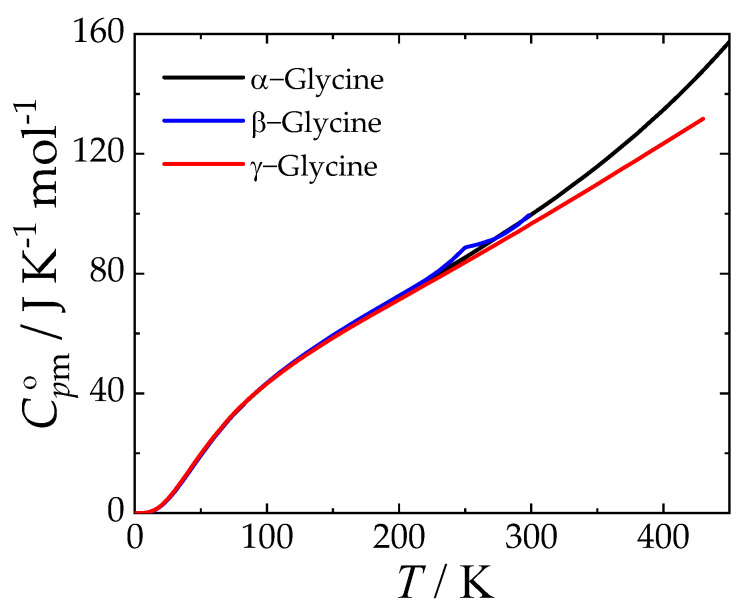
Isobaric heat capacity of stable forms of glycine at *p* = 0.1 MPa.

The standard thermodynamic functions of α-, β-, and γ-glycine obtained using Equations (2) and (3) with parameters from Table 3 are tabulated in Tables S4–S6 in the Supporting Materials. Their values at *T* = 298.15 K are given in Table 4 for convenience. The isobaric heat capacities are compared graphically in Figure 3.

### 2.3. Phase Behavior

Like all other proteinogenic amino acids, α-, β-, and γ-glycine are zwitterionic crystals at 298.15 K; their structures determined by XRPD are provided in Table 5. XRPD diffractograms are shown in Figure S1.

**Figure 4:**
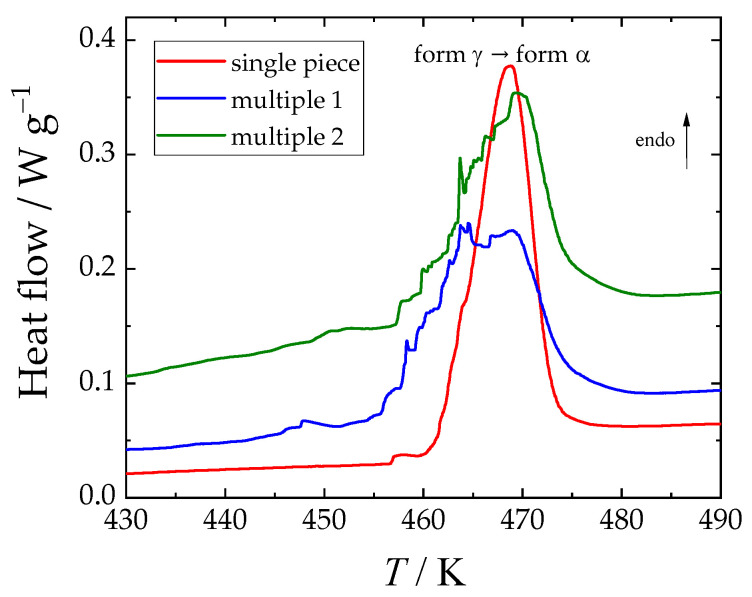
Thermograms obtained using DSC TA Q1000 (TA Instruments, New Castle, DE, USA) for γ-glycine (one piece, 5.83 mg; multiple small pieces, 4.58 mg and 6.37 mg).

The phase behavior of α- and γ-glycine in the temperature range from 183 K to temperatures slightly below the decomposition temperature was investigated by heat-flux DSC. For α-glycine, no phase transition was observed. The γ→α phase transitions observed in this work are shown in Figure 4 and are listed in Table 6 along with the literature values.

The nearly smooth calorimetric peak for the sample consisting of one piece shown in Figure 4 contrasts with the blocky peak for samples of comparable mass consisting of smaller pieces of γ-glycine. The onset temperatures differ by approximately 6 K, with the value for the one-piece sample being higher (see Table 6); however, the enthalpy of this phase transition is (within the uncertainty of measurement) the same. Note that it was found [40] that the temperature of the γ→α phase transition is affected by a number of factors, such as (**a**) the conditions under which the γ-modification crystals are grown, (**b**) the tempering of the crystals prior to the transition experiments, and (**c**) the shape (geometry) of the crystals. For example, Perlovich et al. [40] gave two values *T*_γ→α_ for two fresh samples of different geometries differing by 20 K; these values increased by about 10 K after samples were maintained at an elevated temperature for 5 h. The enthalpy of melting was also influenced by the above factors. Similarly, differences in phase transition between powdered and single-crystal samples were observed (and thoroughly discussed) by Boldyreva et al. [10] and by Tylczyński and Busz [41].

**Table 6:** Phase Transition of γ-Glycine to α-Glycine.

Reference	*T*_γ→α_/K	$\Delta_{\gamma }^{\alpha }H$/kJ mol^−1^	Method	Scanning Rate
1954 Iitaka [14]	438 ± 5	2.5	nosp. ^a^	
1978 Kozhin [42]	438		nosp.	
2001 Perlovich et al. [40]	441.55 ^b^	1.20 ± 0.08 ^b^	PC-DSC	10 K min^−1^
2001 Perlovich et al. [40]	462.45 ^b^	1.80 ± 0.08 ^b^	PC-DSC	10 K min^−1^
2003 Park et al. [43]	450 ^c^		HF-DSC	(1 to 10) K min^−1^
2003 Boldyreva et al. [10] ^d^	422–446 ^e^		HF-DSC ^f^	
2003 Boldyreva et al. [10] ^d^	434–440 ^g^		HF-DSC ^f^	
2005 Yu et al. [44]	396 ^h^	1.9 ^h^	HF-DSC	(1 or 2) K min^−1^
2008 Srinivasan [33]	452.14		TGA	10 K min^−1^
2008 Srinivasan [33]	451.93		DSC	10 K min^−1^
2014 Tylczyński and Busz [41]	460.9 ^e,i^	2.37 ^e,i^	HF-DSC	10 K min^−1^
2014 Tylczyński and Busz [41]	457.6 ^g,i^	2.11 ^g,i^	HF-DSC	10 K min^−1^
2014 Tylczyński and Busz [41]	458		Dielectric constant	0.5 K min^−1^
2014 Tylczyński and Busz [41]	458		Conductivity	0.5 K min^−1^
This work ^j^ small pieces 6.37 mg	455.2 ± 0.3	1.95 ± 0.1	HF-DSC	5 K min^−1^
This work ^j^ small pieces 4.58 mg	456.6 ± 0.3	2.02 ± 0.1	HF-DSC	5 K min^−1^
This work ^j^ single piece 5.83 mg	461.3 ± 0.3	2.04 ± 0.1	HF-DSC	5 K min^−1^

^a^ Method is not specified. Iitaka [14] made the following comment: “The measurement is difficult since the transition speed is rather slow and the temperature at which the transition takes place lies near the decomposition temperature”. ^b^ Perlovich et al. [40] obtained different values at different conditions; see [40] for details. ^c^ Indirect determination from solubility measurements of γ- and α -polymorphs. See [43] for details. ^d^ Boldyreva et al. [10] provided only a graph with Δ$C_{pm}^{o}$ as a function of temperature, from which approximate beginning- and end-of-phase transition peaks can be read. In case of a large single crystal of γ-glycine, four separated peaks with sharp beginnings at 422 K, 431.5 K, 439 K, and 445.5 K were detected. ^e^ γ-Glycine powder. ^f^ Experimental details not provided. In a companion paper [11], it was stated “Calorimetric measurements were performed using DSC-111 (SETARAM), DSC-204 (Netzsch), DSC-30 (Mettler) in the temperature range 140–500 K”. ^g^ Single crystal of γ-glycine. ^h^ Indirect determination from measurements of Gibbs energy difference between crystal polymorphs from their calorimetric data of eutectic melting with a common additive. See [44] for details. ^i^ Values determined from raw data supplied by Tylczyński [45]. ^j^ Values determined in this work are listed together with expanded uncertainties (*k* = 2).

The onset of the γ→α-glycine phase transition reported by Boldyreva et al. [10] ranged from about 422 K to 445 K depending on the form of the sample (small crystal vs. large single crystal vs. powder). While the discussion of the previously published results is thorough, and the lack of substantial experimental details is criticized, Boldyreva et al. [10] did not provide any details on the calorimetric technique used, heating rates, sample masses, or exact phase transition temperatures (the results are presented only in the graphical form).

Tylczyński and Busz [41] prepared a monocrystalline sample of several cubic cm^3^; the powder samples were then prepared by the single-crystal grinding and compressing the powder by the 0.7 GPa pressure. The authors reported the conversion of γ- to α- form by heat-flux DSC and by low-frequency dielectric spectroscopy method on both powder and monocrystalline samples. Differences were found, namely for the powder sample as an observed single DSC peak with onset at 460.9 K, while for the monocrystalline sample, three fused peaks with onset/offset at ca 457.6 K were observed; the enthalpy of the phase transition was, however, comparable (see Table 6). The authors reported that both γ- and α- modifications coexist between 458.5 K and 460 K and stated that the newly formed α- modification is no longer monocrystalline. The onset of signal changes near 458 K was also observed in the real and imaginary parts of the dielectric constants and AC conductivity (the scanning rate was 0.5 K/min in contrast with 10 K/min for the DSC measurements).

The values of this work are also apparently influenced by such factors. While the γ→α transformation appeared at 455–461 K during our measurement using the temperature scanning method (heat-flux DSC TA Q1000), the heat capacity at temperatures above 434 K were apparently already influenced by the γ→α transition. Those heat capacities were measured by the slower method of temperature increments (power-compensation DSC PerkinElmer 8500; see Section 2.2 above).

In fact, this is not a very surprising finding and was already discussed in detail in 1979 by Mnuykh [46] and later, e.g., by Boldyreva et al. [10,11,15] or by Dunitz [47], who provided an interesting discussion and examples of such behavior for other systems. Briefly, solid–solid phase transitions proceed gradually, starting at defects such as surfaces or edges of crystals; after propagating along a given crystal, transition may stop and start at a new defect, possibly at a different temperature [46,47]. Thus, calorimetric measurements are generally unable to determine the true thermodynamic temperature of such a transition.

It can be concluded that the γ→α-glycine phase transition is enantiotropic but irreversible due to kinetic reasons, as discussed, e.g., by Kawakami [48]. While the measured enthalpy change of this work can be considered equal to the enthalpy of the phase transition $\Delta_{\gamma }^{\alpha }H$, all the calorimetric values of phase-transition temperature *T*_γ→α_ (including values obtained in this work) are higher than the equilibrium temperature, which is presumably lower than any calorimetrically determined value.

Table 6 also contains two records obtained by indirect methods. Park et al. [43] attempted to determine the solubility of α- and γ- glycine in water by DSC. Although they reported solubilities up to 442 K, they used only a selection of six data points in the region of about 300 K to 350 K for each polymorph and claimed that their extrapolated intersection at 450 K is the *T*_γ→α_. The choice of data points used was not commented on, and a different selection would lead to different *T*_γ→α_ or would not lead to the intersection at all.

Yu et al. [44] measured the eutectic points of α- and γ- glycine with five different compounds. The highest eutectic temperature (450 K with dulcitol) was excluded, and the remaining four systems were used to derive *T*_γ→α_ = 396 K, which is significantly lower than all other reported values.

Drebushchak et al. [12] attempted to determine the *T*_γ→α_ using the enthalpy of transition derived from solubility data published by Perlovich et al. [40]. The resulting value $\Delta_{\gamma }^{\alpha }H(298.15\;K)$ = 268 ± 105 J mol^−1^ was recalculated to $\Delta_{\gamma }^{\alpha }H\left(0\;K\right)$ = 57 ± 121 J mol^−1^ using their heat capacities obtained by adiabatic calorimetry [12]. As $\Delta_{\gamma }^{\alpha }H(0\;K)=\Delta_{\gamma }^{\alpha }G(0\;K)$, the change in Gibbs energy upon conversion of γ- to α-form can be expressed as

(1) $$\Delta_{\gamma }^{\alpha }G(T)=\Delta_{\gamma }^{\alpha }H(0)+\Delta_{0}^{T}G(\alpha )-\Delta_{0}^{T}G(\gamma )$$

Since the data measured by Drebushchak et al. [12] cover the temperature range (6–305) K, obtaining *T*_γ→α_ would require a long extrapolation (the authors merely mentioned a value 440 K, also given in another paper by the same research group published the same year [11]). With the new heat capacities of this work, which are available up to 434 K, we found that $\Delta_{\gamma }^{\alpha }G=0$ at 415 K; however, due to the considerable uncertainty in $\Delta_{\gamma }^{\alpha }H\left(0\;K\right)$, this transition temperature is associated with significant uncertainty (at least a few tens of kelvins).

## 3. Materials and Methods

### 3.1. Samples Description

Glycine of commercial origin was found to be α-polymorph; it was used as received. The γ-polymorph was prepared following the method described by Srinivasan [33], with modifications to obtain γ-polymorph crystals free from NaCl contamination. The α-polymorph was gradually dissolved in the aqueous solution of NaCl (8.5 g NaCl per 100 mL of distilled water) at 333 K. The solution was then slowly cooled to the room temperature. The resulting crystals were filtered and washed with an aqueous solution of glycine to remove any NaCl residue. After drying at 313 K for one day, the crystals were analyzed by XRPD, confirming the γ-polymorph crystalline form without NaCl contamination. The purities of the samples used for measurements in this work are reported in Table 7.

### 3.2. Thermogravimetry

Prior to assessing heat capacity and phase behavior, thermogravimetric analysis (TGA) was conducted. This was performed using the SETARAM Setsys Evolution (SETARAM, Caluire-et-Cuire, France) thermogravimetric analyzer. Each sample was placed in a 100 μL platinum crucible in an open configuration. The analysis spanned a temperature range from 298 K to 573 K, with a rate of temperature increase set to 5 K min^−1^ under an argon atmosphere to maintain an inert environment.

### 3.3. Heat Capacity Measurements

Heat capacities were measured using a SETARAM μDSC IIIa (SETARAM, Caluire-et-Cuire, France) calorimeter of the Tian–Calvet type, covering a temperature span from 262 K to 358 K. As the calorimeter’s design, calibration, and operation were discussed in detail in earlier studies [1], only the essential information is presented here. The measurements followed a continuous heating approach [49] and a three-step procedure: measurements on the sample, then on a synthetic sapphire reference (NIST Standard No. 720) [50], and finally a blank experiment. Given the very low sublimation pressures of the samples, the saturated molar heat capacities $C_{\mathrm{sat}}$ derived here are equivalent to isobaric molar heat capacities $C_{pm}^{o}$ over this temperature range. The combined expanded uncertainty (0.95 level of confidence) of the heat capacity measurements is estimated to be $U_{c}(C_{pm}^{o})=0.01\cdot C_{pm}^{o}$. For additional heat capacity measurements in the 309–449 K range, a PerkinElmer DSC 8500 (PerkinElmer, Waltham, MA, USA) with a power compensation system and an autosampler was employed. Data for γ-glycine above 434 K were excluded from the fit due to the onset of the γ→α phase transition near 455 K. These measurements used a temperature increment approach, repeated thrice to reduce systematic errors. The combined expanded uncertainty (0.95 level of confidence) of the heat capacity measurement was estimated to be $U_{c}(C_{pm}^{o})=0.03\cdot C_{pm}^{o}$. To account for minor discrepancies with the more precise SETARAM μDSC IIIa results, scaling adjustments of 0.98 and 0.973 were applied to α-glycine and γ-glycine, respectively, in line with common practice [51]. Both corrected and raw values are included in Supporting Materials (Tables S1 and S2). No scaling was necessary in case of low-temperature relaxation calorimetry, described in the next paragraph (see Supporting Materials Table S3).

Low-temperature heat capacity measurements of α-glycine were performed using a Physical Property Measurement System (PPMS) Model 6000 EverCool II (Quantum Design, San Diego, CA, USA) equipped with a heat capacity module (^4^He, *T*_min_ = 1.8 K). This calorimeter uses a thermal-relaxation method that serves as an efficient alternative to traditional adiabatic calorimetry. The specific heat capacities were obtained by tracking the thermal response to a change in heating conditions [52]. The measurement procedure was similar to that described by Shi et al. [53], but the samples were not mixed with Apiezon N, as in [53]. Instead, the samples placed in 3.5 mm × 3 mm copper cups (made from 0.025 mm copper foil, purity 99.999%, provided by Alfa Aesar, Ward Hill, MA, USA) were compressed to a height of approximately 1 mm using a stainless-steel die (Maassen, Ludwigsburg, Germany) and a press (Trystom, Olomouc, Czech Republic). The heat capacity of the copper cup was subtracted from the total heat capacity based on the recommended data from Arblaster [54]. This technique was previously validated [2,55] by measuring compounds with reliable data obtained by adiabatic calorimetry (anthracene [56] and L-asparagine [57]) and provides uncertainties comparable to a thoroughly tested method [23] employing hermetic aluminum DSC pans for sample encapsulation. The results of this work for α-glycine confirm the reliability of the technique using Cu cups. The combined expanded uncertainty (0.95 level of confidence) of the heat capacity measurements was estimated to be $U_{c}(C_{pm}^{o})=0.10\cdot C_{pm}^{o}$ below 10 K, $U_{c}(C_{pm}^{o})=0.03\cdot C_{pm}^{o}$ in the temperature range (10 to 40) K, and $U_{c}(C_{pm}^{o})=0.02\cdot C_{pm}^{o}$ in the temperature range (40 to 300) K. Deviations from adiabatic calorimetry values [12] fall within these bounds (see Figure 2a). Although the uncertainty of PPMS results was larger than that of the adiabatic calorimetry results, they were included in the data fitting because they extended the temperature range from 7 K [12] down to 2 K.

To represent the temperature dependence of heat capacity over a wide range of temperatures (including the literature heat capacities obtained by adiabatic calorimetry and data obtained by Quantum Design PPMS), we employed the equation proposed by Archer [58]:

(2) $$C_{pm}^{o}/C_{pm}^{\mathrm{ref}}={\left(\frac{T}{T^{\mathrm{ref}}f(T)+bT}\right)}^{3}$$

where $T^{\mathrm{ref}}$ = 1 K, and $C_{pm}^{\mathrm{ref}}$ = 1 J K^−1^ mol^−1^, and

(3) $$f(T)=a_{i}{\left(T-T_{i}\right)}^{3}+b_{i}{\left(T-T_{i}\right)}^{2}+c_{i}\left(T-T_{i}\right)+d_{i}$$

where only a single parameter *d_i_* per each temperature interval is to be optimized, while the values of the other three are imposed by a constraint of continuity and smoothness of the resulting temperature dependence. Parameter *b* is estimated from the slope of f(T) at temperatures greater than 70 K prior to the optimization procedure [58].

### 3.4. Phase Behavior Measurements

X-ray powder diffraction (XRPD) was utilized to determine initial crystal structures of the α-glycine and γ-glycine. Measurements were conducted using a *θ*–*θ* powder diffractometer X’Pert3 Powder (PANalytical, Malvern, UK) in Bragg–Brentano para-focusing geometry using wavelength CuKα radiation (*λ* = 1.5418 Å, *U* = 40 kV, *I* = 30 mA). Sample scans were performed at 298.15 K using standard settings (the range of 5° to 50° 2*θ* with a step size of 0.039° 2*θ* and 0.7 s per step). The resulting diffractograms were analyzed using HighScore Plus software v5.1 [59], supported by annually updated powder diffraction databases PDF4+ and PDF4/Organics [60].

To explore the phase behavior of α- and γ-glycine over a temperature range from 183 K up to the decomposition temperature, a TA Instruments Q1000 heat-flux differential scanning calorimeter (DSC) was employed. The combined expanded uncertainty (0.95 level of confidence) in the phase transition temperatures and enthalpies are 0.3 K and 0.1 kJ mol^−1^, respectively.

## 4. Conclusions

Temperature range over which the heat capacities for α-glycine and γ-glycine are known was extended using a combination of two different calorimetric techniques (Tian–Calvet, PC DSC); α-glycine was also studied using relaxation calorimetry. Accurate equations for the crystal heat capacity in the temperature range from 0 K to 450 K (α-glycine) and to 435 K (γ-glycine) were established. These equations are based on the new heat capacity measurements performed in this work and also on low-temperature adiabatic heat capacity data from the literature. Standard thermodynamic functions (entropy, enthalpy, and Gibbs energy) of the crystalline phase at *p* = 0.1 MPa were evaluated within the range of validity of the heat capacity equations (see Tables S4–S6 in the Supplementary Materials). The same fitting procedure was applied for the literature heat capacities of β-glycine over the temperature range from 0 to 295 K.

The decomposition temperature of the α-glycine (512 K) was determined by TGA. The phase behavior was studied by heat-flux differential scanning calorimetry over a temperature range from 183 K to temperatures slightly below the decomposition temperature to determine the presence of possible phase transitions. No phase transition was detected for α-glycine, while γ-glycine was transformed to α-glycine at 455–461 K, which is apparently above the true thermodynamic temperature *T*_γ→α_. The enthalpy of this phase transition was determined. The actual thermodynamic temperature *T*_γ→α_ = 415 K was determined according to the previously described procedure and taking advantage of the new heat capacities of this work.

## Figures and Tables

**Table 2:** Overview of the Literature Crystal Heat Capacities of α-, *β*-, and γ-Glycine.

Reference ^a^	*N* ^b^	(*T*_min_–*T*_max_)/K	$100\;u_{r}(C_{p.m}^{\mathrm{cr}})$ ^c^	Method
α-glycine				
Parks et al. [27] ^d^	16	93–299	1.0	adiabatic
Hutchens et al. [28] ^d^	81	12–302	0.4	adiabatic
Spink and Wadsö [35] ^d^	1	298	0.1	drop
Drebushchak et al. [36]	S	310–340	nosp.	HF DSC ^e^
**Drebushchak et al.** [12]	**62**	**7–304**	**0.5**	**adiabatic**
**This work**	**21**	**262–358**	**1.0**	**Tian–Calvet**
**This work**	**116**	**2–267**	**^f^**	**relaxation**
**This work**	**29**	**310–449**	**3.0**	**PC DSC ^e^**
β-glycine				
**Drebushchak et al.** [9]	**112**	**6–295**	**0.5**	**adiabatic**
γ-glycine				
**Drebushchak et al.** [12]	**66**	**6–305**	**0.5**	**adiabatic**
Badelin et al. [37] ^d^	4	298–348	1.0	HF DSC ^e^
**This Work**	**21**	**262–358**	**1.0**	**Tian-Calvet**
**This Work**	**27**	**305–434**	**3.0**	**PC DSC ^e^**

^a^ The data from references written in bold were fitted to Equations (1) and (2). ^b^ *N* = number of data points. S denotes smoothed data in the form of equation. ^c^ *u*_r_ ($C_{pm}^{o}$) stands for relative uncertainty in heat capacity as stated by the authors. Nosp. stands for not specified. ^d^ Polymorph not explicitly specified. Polymorph deduced from the values of heat capacity. ^e^ HF DSC stands for heat-flux DSC, and PC DSC stands for power-compensation DSC. ^f^ For specification of *u*_r_ ($C_{pm}^{o}$) of PPMS using thermal relaxation measurement technique, see Section 3.3.

**Table 3:** Parameters of Equations (2) and (3) for Glycine Heat Capacities in J K^−1^ mol^−1^.

*a*_i_/K^−3^	*b*_i_/K^−2^	*c*_i_/K^−1^	*d*_i_	*T*_i_/K	*T*_i+1_/K	*N* ^a^	*s*_r_^b^
		α-glycine		*b*^c^ = 0.17			
−2.84804 × 10^−2^	3.79575 × 10^−1^	−1.72989 × 10^0^	1.80581 × 10^1^	0	5	12	1.66
2.65771 × 10^−3^	−4.76310 × 10^−2^	−7.01746 × 10^−2^	1.53379 × 10^1^	5	13	16	2.87
−3.60012 × 10^−4^	1.61540 × 10^−2^	−3.21990 × 10^−1^	1.30889 × 10^1^	13	25	19	1.51
−3.40067 × 10^−5^	3.19356 × 10^−3^	−8.98193 × 10^−2^	1.09291 × 10^1^	25	50	22	1.05
−4.72203 × 10^−6^	6.43060 × 10^−4^	6.09624 × 10^−3^	1.01482 × 10^1^	50	100	31	0.43
−3.02689 × 10^−7^	−6.52444 × 10^−5^	3.49870 × 10^−2^	1.14704 × 10^1^	100	200	44	0.20
−3.78620 × 10^−8^	−1.56051 × 10^−4^	1.28575 × 10^−2^	1.40140 × 10^1^	200	450	84	0.18
		β-glycine		*b*^c^ = 0.17			
−1.45493 × 10^−2^	7.27466 × 10^−2^	1.08677 × 10^0^	7.71257 × 10^0^	0	5	0	0.00
6.70899 × 10^−3^	−1.45493 × 10^−1^	7.23038 × 10^−1^	1.31464 × 10^1^	5	13	14	3.63
−3.39735 × 10^−4^	1.55225 × 10^−2^	−3.16728 × 10^−1^	1.30542 × 10^1^	13	25	8	1.50
−3.56913 × 10^−5^	3.29205 × 10^−3^	−9.09534 × 10^−2^	1.09016 × 10^1^	25	50	9	0.16
−4.55449 × 10^−6^	6.15203 × 10^−4^	6.72786 × 10^−3^	1.01276 × 10^1^	50	100	12	0.16
−2.21122 × 10^−7^	−6.79704 × 10^−5^	3.40895 × 10^−2^	1.14327 × 10^1^	100	200	14	0.16
−5.95660 × 10^−6^	−1.34307 × 10^−4^	1.38617 × 10^−2^	1.39408 × 10^1^	200	252	24	0.25
-	−9.73021 × 10^−4^	4.70409 × 10^−2^	1.35535 × 10^1^	252	295	31	0.61
		γ-glycine		*b*^c^ = 0.17			
9.25051 × 10^−3^	−4.62526 × 10^−2^	−1.25048 × 10^0^	2.26389 × 10^1^	0	5	0	0.00
−3.53099 × 10^−3^	9.25051 × 10^−2^	−1.01922 × 10^0^	1.63865 × 10^1^	5	13	6	4.98
−1.25469 × 10^−4^	7.76139 × 10^−3^	−2.17085 × 10^−1^	1.23453 × 10^1^	13	25	13	0.57
−3.53314 × 10^−5^	3.24451 × 10^−3^	−8.50141 × 10^−2^	1.06411 × 10^1^	25	50	9	0.18
−4.42334 × 10^−6^	5.94658 × 10^−4^	1.09651 × 10^−2^	9.99148 × 10^0^	50	100	10	0.15
−2.77082 × 10^−7^	−6.88432 × 10^−5^	3.72558 × 10^−2^	1.14735 × 10^1^	100	200	13	0.11
1.41907 × 10^−7^	−1.51968 × 10^−4^	1.51747 × 10^−2^	1.42335 × 10^1^	200	435	63	0.27

^a^ *N* stands for number of experimental data points in given temperature interval used for correlation. ^b^ $s_{r}=100{\left\{\sum_{i=1}^{n}{\left[\left(C_{pm}^{\exp}-C_{pm}^{o}\right)/C_{pm}^{o}\right]}_{i}^{2}/\left(N-m\right)\right\}}^{1/2}$, where $C_{pm}^{\exp}$ and $C_{pm}^{o}$ are the experimental and calculated (Equations (2) and (3)) heat capacity, *N* is the number of fitted data points, and *m* is the number of independent adjustable parameters. ^c^ Parameter *b* from Equation (2).

**Table 4:** Standard Thermodynamic Functions of Stable Forms of Glycine at *p* = 0.1 MPa and *T* = 298.15 K ^a^.

Compound	$C_{pm}^{o}$/J K^−1^ mol^−1^	$S_{m}^{o}$/J K^−1^ mol^−1^	$\Delta_{0}^{T}H_{m}^{o}$/kJ mol^−1^	$\Delta_{0}^{T}G_{m}^{o}$/kJ mol^−1^
α-glycine	99.13	103.7	16.18	−14.74
β-glycine ^b^	99.52	104.3	16.29	−14.82
γ-glycine	96.16	103.4	15.99	−14.83

^a^ The combined expanded uncertainty of heat capacity $U_{c}(C_{pm}^{o})$ as well as of all calculated thermodynamic values (with 0.95 level of confidence, *k* = 2) is *U*_c_(*X*) = 0.01 *X* at 298.15 K, where *X* represents the heat capacity or the thermodynamic property. Values are reported with one digit more than is justified by the experimental uncertainty to avoid round-off errors in calculations based on these results. ^b^ Extrapolated from 295 K using Equations (2) and (3) with parameters from Table 3.

**Table 5:** Normal-Pressure Crystal Structures of Glycine Polymorphs.

Compound	Refcode ^a^	*Z*	Space Group	Ref. ^b^
α-glycine	GLYCIN02	4	*P*2_1_/n	Marsh [38]
β-glycine	GLYCIN	2	*P*2_1_	Iitaka [39]
γ-glycine	GLYCIN01	3	*P*3_2_	Iitaka [14]

^a^ Identifier in the Cambridge Structural Database (CSD). ^b^ Reference in which crystal structure parameters with a given Refcode were determined.

**Table 7:** Sample Description.

Compound	CAS RN	Supplier	Mole Fraction Purity ^a^	Purity Method ^a^
α-Glycine	56-40-6	Sigma-Aldrich (St. Louis, MO, USA)	1.00 ^b^	HPLC
γ-Glycine	56-40-6	Fischer (Achern-Fautenbach, Germany)	1.00 ^b^	HPLC

^a^ From certificate of analysis supplied by the manufacturer. ^b^ No impurities were identified.

## Data Availability

The data presented in this study are available in the Supplementary Materials.

## Supplementary Materials

The following supporting information can be downloaded at https://www.mdpi.com/article/10.3390/molecules29225366/s1, Figure S1: XRPD diffractograms for α-glycine and γ-glycine, Table S1: Experimental molar heat capacity of α-glycine, Table S2: Experimental molar heat capacity of γ-glycine, Table S3: Experimental molar heat capacity of α-glycine obtained using the relaxation technique (Quantum Design PPMS), Table S4: Standard thermodynamic functions of α-glycine at p = 0.1 MPa., Table S5: Standard thermodynamic functions of β-glycine at p = 0.1 MPa., Table S6: Standard thermodynamic functions of γ-glycine at p = 0.1 MPa.
