# Supplementary material for: Heat Capacities of α-, β-, and γ- Polymorphs of Glycine
Source: Molecules. 2024 Nov 14;29(22):5366. doi: 10.3390/molecules29225366 (PMC11596786; doi:10.3390/molecules29225366)
Supplement: Supplementary file 1 [file molecules-29-05366-s001.zip › molecules-3304376-supplementary.pdf]

molecules

MDPI

Supplementary Materials

Heat capacities of α-, β-, and γ- polymorphs of glycine

Václav Pokorný [1,2], Vojtěch Štejfa [1], Jakub Havlín [3], Michal Fulem [1], and Květoslav Růžička [1,*]

1 Department of Physical Chemistry, University of Chemistry and Technology, Prague, Technická 5, CZ-166 28 Prague, Czech Republic; pokorny@imc.cas.cz (V.P.); stejfav@vscht.cz (V.Š.); fulemm@vscht.cz (M.F.)
2 Institute of Macromolecular Chemistry, Czech Academy of Sciences, Heyrovského nám. 2, CZ-162 06 Prague, Czech Republic

3 Central Laboratories, University of Chemistry and Technology, Prague, Technická 5, CZ-166 28 Prague, Czech Republic; havlinj@vscht.cz

* Correspondence: ruzickak@vscht.cz

Supplementary materials contain the following:

1) XPRD diffractograms for α-glycine and γ-glycine.
2) Experimental heat capacity data for α-glycine and γ-glycine measured using SETARAM μDSC IIIa, PerkinElmer DSC 8500, and Quantum Design PPMS (Tables S1 to S3).
3) Tabulated thermodynamic functions (heat capacity, entropy, enthalpy, Gibbs energy) of α-glycine, β-glycine, and γ-glycine (Tables S4 to S6).

1) XRPD diffractograms for α-glycine and γ-glycine

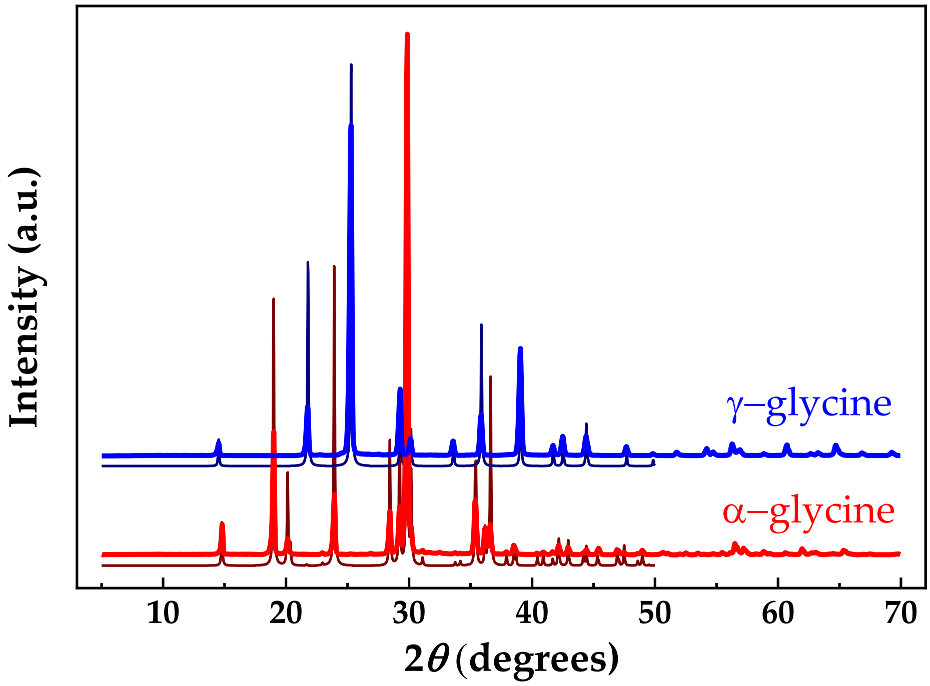

Figure S1. XRPD diffractograms for α-glycine and γ-glycine. Dark thin lines show simulated curves from the Cambridge Structural Database.

2) Experimental heat capacities

Table S1. Experimental molar heat capacity C^{o}_{pm} of α-glycine (in J K^{-1} mol^{-1}) at p = (100 ± 5) kPa.

SETARAM μDSC IIIa[a] (*m* = 613.00 mg)			PerkinElmer DSC 8500[b] (*m* = 18.28 mg)			
T / K	C^{o}_{pm} / J·K^{-1}·mol^{-1} [c]	100δ$_{rel}$ [d]	T / K	C^{o}_{pm} / J·K^{-1}·mol^{-1} [c]	C^{o}_{pm} corrected [e]	100δ$_{rel}$ [d]
262.38	88.68	-0.18	309.88	105.36	102.54	-0.16
265.00	89.54	-0.02	314.84	106.95	104.09	-0.14
270.00	91.01	0.06	319.81	108.69	105.78	-0.02
275.00	92.45	0.08	324.77	110.44	107.49	0.09
280.00	93.82	0.02	329.74	111.95	108.95	-0.04
285.00	95.15	-0.10	334.71	113.75	110.71	0.07
290.00	96.56	-0.15	339.68	115.36	112.28	-0.02
295.00	98.11	-0.07	344.65	117.18	114.05	0.06
300.00	99.66	-0.02	349.62	118.86	115.68	0.00
305.00	101.19	-0.01	354.6	120.55	117.33	-0.08
310.00	102.72	-0.01	359.57	121.89	118.63	-0.47
315.00	104.32	0.03	364.55	123.99	120.67	-0.26
320.00	105.91	0.05	369.52	125.82	122.45	-0.28
325.00	107.51	0.04	374.5	127.68	124.26	-0.31
330.00	109.12	0.03	379.47	129.51	126.04	-0.39
335.00	110.71	-0.02	384.45	131.54	128.02	-0.34
340.00	112.31	-0.08	389.43	133.79	130.21	-0.15
345.00	114.01	-0.07	394.41	135.84	132.21	-0.13
350.00	115.88	0.05	399.4	138.15	134.46	0.04
355.00	117.67	0.09	404.38	140.29	136.54	0.06
358.00	118.71	0.08	409.37	142.70	138.88	0.23
			414.36	145.12	141.23	0.38
			419.34	147.37	143.43	0.39
			424.33	149.59	145.59	0.35
			429.32	151.98	147.92	0.39
			434.31	154.52	150.39	0.49
			439.29	156.78	152.58	0.39
			444.28	159.27	155.01	0.41
			449.26	161.07	156.76	-0.03

[a] Standard uncertainty of temperature is $u(T)$ = 0.05 K, and the combined expanded uncertainty of the heat capacity is $U_c(C^{o}_{pm}) = 0.01\, C^{o}_{pm}$ (0.95 level of confidence).
[b] Standard uncertainty of temperature is $u(T)$ = 0.05 K, and the combined expanded uncertainty of the heat capacity is $U_c(C^{o}_{pm}) = 0.03\, C^{o}_{pm}$ (0.95 level of confidence).
[c] Values are reported with more digits than is justified by the experimental uncertainty to avoid round-off errors in calculations based on these results.

[d] $\delta_{\mathrm{rel}} = \left(C_{pm}^{\mathrm{o,exp}} - C_{pm}^{\mathrm{o,calc}}\right) / C_{pm}^{\mathrm{o,calc}}$, where $C_{pm}^{\mathrm{o,calc}}$ is heat capacity calculated by means of Eqs. 2 and 3 with parameters from Table 3.

[e] Experimental heat capacity data from PerkinElmer DSC 8500 has been multiplied by the factor of 0.973 to agree with the more accurate heat capacities obtained with SETARAM μDSC IIIa data.

Table S2. Experimental molar heat capacity C_{pm}^{o} of γ-glycine (in J K^{-1} mol^{-1}) at p = (100 ± 5) kPa.

SETARAM μDSC IIIa[a] (m = 371.41 mg)			PerkinElmer DSC 8500[b] (m = 26.97 mg)			
T / K	C_{pm}^{o} / J·K^{-1}·mol^{-1} [c]	100δ_{rel} [d]	T / K	C_{pm}^{o} / J·K^{-1}·mol^{-1} [c]	C_{pm}^{o} corrected [e]	100δ_{rel} [d]
261.91	86.88	0.07	304.96	99.93	97.97	0.03
265.00	87.64	0.04	309.92	101.31	99.32	0.08
270.00	88.83	-0.06	314.88	102.64	100.62	0.08
275.00	90.22	0.05	319.84	104.11	102.06	0.20
280.00	91.49	0.04	324.81	105.50	103.43	0.25
285.00	92.81	0.07	329.77	106.69	104.59	0.10
290.00	94.18	0.15	334.74	107.88	105.76	-0.05
295.00	95.52	0.19	339.71	109.27	107.13	-0.01
300.00	96.88	0.25	344.69	110.62	108.45	-0.02
305.00	98.32	0.38	349.66	111.98	109.78	-0.03
310.00	99.72	0.46	354.64	113.56	111.33	0.16
315.00	101.01	0.43	359.61	115.16	112.89	0.36
320.00	102.39	0.48	364.59	116.83	114.54	0.61
325.00	103.76	0.52	369.57	118.17	115.85	0.57
330.00	104.96	0.39	374.54	119.57	117.22	0.58
335.00	106.17	0.28	379.52	120.28	117.91	0.02
340.00	107.34	0.11	384.5	121.68	119.29	0.03
345.00	108.33	-0.21	389.48	123.22	120.80	0.15
350.00	109.61	-0.26	394.46	124.85	122.39	0.35
355.00	110.92	-0.29	399.44	125.65	123.18	-0.12
358.31	111.74	-0.35	404.43	127.35	124.84	0.11
			409.42	128.71	126.18	0.09
			414.41	130.20	127.64	0.16
			419.39	132.30	129.70	0.69
			424.38	132.67	130.06	-0.09
			429.37	134.62	131.97	0.32
			434.36	134.72	132.07	-0.64

[a,b,c,d] For footnote explanation see Table S1

[e] Experimental heat capacity data from PerkinElmer DSC 8500 has been multiplied by the factor of 0.980 to agree with the more accurate heat capacities obtained with SETARAM μDSC IIIa data.

Table S3. Experimental molar heat capacity C_{pm}^{o} of α-glycine obtained using the relaxation technique (Quantum Design PPMS).[a] m_{sample} = 20.358 mg, m_{Cu} = 15.259 mg.

T / K	C_{pm}^{o} / J·K^{-1}·mol^{-1}	100δ_{rel} [b]	T / K	C_{pm}^{o} / J·K^{-1}·mol^{-1}	100δ_{rel} [b]
2.03	0.001994	2.22	80.70	35.65	0.04
2.03	0.001991	1.94	80.70	35.59	-0.13
2.42	0.003313	-2.02	85.74	37.98	0.48
2.42	0.003336	-1.37	85.74	38.11	0.82
2.88	0.005675	-2.30	90.77	40.17	0.82
2.88	0.005670	-2.40	90.78	40.20	0.90
3.43	0.009850	0.55	95.81	42.15	0.88
3.43	0.009833	0.38	95.81	41.95	0.40
4.09	0.01643	0.18	100.84	43.83	0.44
4.09	0.01647	0.41	100.85	43.66	0.04
4.85	0.02710	0.36	106.90	45.89	0.23
4.85	0.02745	1.63	106.90	46.02	0.51
5.77	0.04577	2.23	113.88	48.09	-0.13
5.77	0.04582	2.33	113.88	48.35	0.41
6.86	0.07711	2.20	120.86	50.35	-0.14
6.86	0.07674	1.67	120.86	50.31	-0.22
8.11	0.1310	2.84	127.85	52.50	-0.19
8.11	0.1301	2.07	127.85	52.54	-0.12
10.13	0.2706	2.95	134.83	54.78	0.14
10.13	0.2697	2.61	134.83	54.79	0.16
12.14	0.4957	2.00	141.81	56.92	0.32
12.14	0.4966	2.19	141.81	56.81	0.13
14.16	0.8313	-6.35	148.76	58.84	0.22
14.16	0.8299	-6.53	148.76	58.73	0.04
16.17	1.289	-2.36	155.74	60.48	-0.26
16.18	1.283	-2.84	155.75	60.57	-0.12
18.19	1.849	-0.59	162.73	62.59	0.09
18.19	1.849	-0.60	162.73	62.35	-0.30
20.21	2.530	0.89	169.72	64.34	-0.09
20.21	2.533	0.99	169.72	64.46	0.10
22.23	3.312	1.38	176.73	66.22	-0.03
22.23	3.311	1.35	176.73	66.05	-0.29
24.25	4.209	2.05	183.71	68.25	0.28
24.25	4.212	2.11	183.71	68.03	-0.05
26.26	5.174	2.01	190.67	70.00	0.19
26.26	5.181	2.15	190.67	69.94	0.11

T / K	C^{o}_{pm} / J·K^{-1}·mol^{-1}	100δ_{rel} [b]	T / K	C^{o}_{pm} / J·K^{-1}·mol^{-1}	100δ_{rel} [b]
28.28	6.211	1.80	197.64	71.84	0.24
28.28	6.200	1.62	197.64	71.89	0.31
30.30	7.303	1.41	204.60	73.64	0.24
30.30	7.304	1.43	204.60	73.50	0.05
35.33	10.27	1.04	211.56	75.67	0.53
35.34	10.28	1.12	211.56	75.51	0.32
40.37	13.38	0.66	218.52	77.30	0.29
40.37	13.39	0.73	218.52	77.43	0.46
45.42	16.53	0.50	225.50	78.87	-0.05
45.42	16.53	0.49	225.50	78.87	-0.05
50.45	19.71	0.82	232.47	80.70	-0.05
50.45	19.71	0.81	232.47	80.85	0.13
55.49	22.69	0.51	239.43	82.46	-0.16
55.49	22.70	0.55	239.44	82.54	-0.07
60.53	25.50	0.06	246.41	84.46	-0.01
60.53	25.52	0.14	246.41	84.16	-0.36
65.58	28.29	0.11	253.38	86.43	0.08
65.58	28.27	0.03	253.38	86.40	0.05
70.62	30.88	0.02	260.34	88.35	0.09
70.62	30.89	0.05	260.34	88.17	-0.11
75.66	33.36	0.09	267.30	90.30	0.11
75.67	33.27	-0.18	267.30	90.37	0.19

[a] Standard uncertainty of temperature is $u(T)$ = 0.004 K, and the combined expanded uncertainty of heat capacity $U_c(C^{o}_{pm})$ with 0.95 level of confidence (k = 2) is $U_c(C^{o}_{pm})$ = 0.1 C^{o}_{pm} below 10 K; $U_c(C^{o}_{pm})$ = 0.03 C^{o}_{pm} in temperature range (10 to 40) K; $U_c(C^{o}_{pm})$ = 0.02 C^{o}_{pm} in temperature range (40 to 300) K. Values are reported with more digits than is justified by the experimental uncertainty to avoid round-off errors in calculations based on these results. Measurements are performed in vacuum (residual pressure $p<10^{-4}$ Pa).

[b] $\delta_{rel} = \left(C^{o,exp}_{pm} - C^{o,calc}_{pm}\right) / C^{o,calc}_{pm}$, where $C^{o,calc}_{pm}$ is heat capacity calculated by means of Eqs. 2 and 3 with parameters from Table 3.

3) Tabulated thermodynamic functions

Standard thermodynamic functions were calculated using fundamental thermodynamic relationships (assuming residual entropy at 0 K for all crystalline forms of glycine to be 0 J·K⁻¹·mol⁻¹) and heat capacities $C^{\mathrm{o}}_{p\mathrm{m}}(T)$ represented by Eqs 2 and 3 using parameters listed in Table 3:

$$S^{\mathrm{o}}_{\mathrm{m}}(T) = \int_0^T \frac{C^{\mathrm{o}}_{p\mathrm{m}}(T)}{T}\,\mathrm{d}T \tag{S1}$$

$$\Delta_0^T H^{\mathrm{o}}_{\mathrm{m}} = \int_0^T C^{\mathrm{o}}_{p\mathrm{m}}(T)\,\mathrm{d}T \tag{S2}$$

$$\Delta_0^T G^{\mathrm{o}}_{\mathrm{m}} = \Delta_0^T H^{\mathrm{o}}_{\mathrm{m}} - T S^{\mathrm{o}}_{\mathrm{m}}(T) \tag{S3}$$

Table S4. Standard thermodynamic functions of α-glycine[a] at p = 0.1 MPa.[b]

T / K	$C^{\mathrm{o}}_{p\mathrm{m}}$ / J·K⁻¹·mol⁻¹	$S^{\mathrm{o}}_{\mathrm{m}}$ / J·K⁻¹·mol⁻¹	$\Delta_0^T H^{\mathrm{o}}_{\mathrm{m}}$ / kJ·mol⁻¹	$\Delta_0^T G^{\mathrm{o}}_{\mathrm{m}}$ / kJ·mol⁻¹
1[c]	2.091×10^{-4}	6.671×10^{-5}	5.050×10^{-8}	-1.621×10^{-8}
2[c]	1.872×10^{-3}	5.930×10^{-4}	9.005×10^{-7}	-2.855×10^{-7}
3	6.560×10^{-3}	2.115×10^{-3}	4.809×10^{-6}	-1.535×10^{-6}
4	1.543×10^{-2}	5.105×10^{-3}	1.542×10^{-5}	-5.006×10^{-6}
5	2.947×10^{-2}	9.947×10^{-3}	3.737×10^{-5}	-1.236×10^{-5}
6	5.041×10^{-2}	1.705×10^{-2}	7.665×10^{-5}	-2.565×10^{-5}
7	8.040×10^{-2}	2.694×10^{-2}	1.412×10^{-4}	-4.739×10^{-5}
8	1.220×10^{-1}	4.025×10^{-2}	2.413×10^{-4}	-8.067×10^{-5}
9	1.781×10^{-1}	5.770×10^{-2}	3.900×10^{-4}	-1.293×10^{-4}
10	2.522×10^{-1}	8.013×10^{-2}	6.036×10^{-4}	-1.977×10^{-4}
11	3.475×10^{-1}	1.085×10^{-1}	9.015×10^{-4}	-2.915×10^{-4}
12	4.673×10^{-1}	1.436×10^{-1}	1.307×10^{-3}	-4.169×10^{-4}
13	6.136×10^{-1}	1.866×10^{-1}	1.845×10^{-3}	-5.814×10^{-4}
14	7.872×10^{-1}	2.383×10^{-1}	2.543×10^{-3}	-7.931×10^{-4}
15	9.888×10^{-1}	2.993×10^{-1}	3.429×10^{-3}	-1.061×10^{-3}
16	1.219	3.703×10^{-1}	4.530×10^{-3}	-1.395×10^{-3}
17	1.477	4.518×10^{-1}	5.876×10^{-3}	-1.805×10^{-3}
18	1.764	5.442×10^{-1}	7.494×10^{-3}	-2.302×10^{-3}
19	2.077	6.479×10^{-1}	9.412×10^{-3}	-2.897×10^{-3}
20	2.417	7.630×10^{-1}	1.166×10^{-2}	-3.602×10^{-3}
25	4.468	1.512	2.864×10^{-2}	-9.169×10^{-3}
30	7.037	2.547	5.721×10^{-2}	-1.920×10^{-2}
35	9.962	3.848	9.959×10^{-2}	-3.508×10^{-2}
40	13.06	5.379	1.571×10^{-1}	-5.806×10^{-2}
45	16.19	7.099	2.302×10^{-1}	-8.919×10^{-2}

T / K	C^{o}_{pm} / J·K^{-1}·mol^{-1}	S^{o}_{m} / J·K^{-1}·mol^{-1}	$\Delta^{T}_{0}H^{o}_{m}$ / kJ·mol^{-1}	$\Delta^{T}_{0}G^{o}_{m}$ / kJ·mol^{-1}
50	19.28	8.965	3.189×10^{-1}	-1.293×10^{-1}
55	22.29	10.94	4.229×10^{-1}	-1.790×10^{-1}
60	25.19	13.01	5.416×10^{-1}	-2.389×10^{-1}
65	27.95	15.13	6.745×10^{-1}	-3.092×10^{-1}
70	30.56	17.30	8.209×10^{-1}	-3.903×10^{-1}
75	33.02	19.50	9.799×10^{-1}	-4.823×10^{-1}
80	35.32	21.70	1.151	-5.853×10^{-1}
85	37.49	23.91	1.333	-6.993×10^{-1}
90	39.54	26.11	1.526	-8.243×10^{-1}
95	41.48	28.30	1.728	-9.603×10^{-1}
100	43.33	30.47	1.940	-1.107
110	46.85	34.77	2.391	-1.434
120	50.15	38.99	2.876	-1.802
130	53.26	43.13	3.394	-2.213
140	56.22	47.18	3.941	-2.665
150	59.06	51.16	4.517	-3.157
160	61.80	55.06	5.122	-3.688
170	64.47	58.89	5.753	-4.257
180	67.10	62.65	6.411	-4.865
190	69.69	66.34	7.095	-5.510
200	72.28	69.98	7.805	-6.192
210	74.86	73.57	8.541	-6.910
220	77.47	77.11	9.302	-7.663
230	80.09	80.62	10.09	-8.452
240	82.75	84.08	10.90	-9.275
250	85.44	87.51	11.75	-10.13
260	88.17	90.92	12.61	-11.03
270	90.96	94.30	13.51	-11.95
273.15	91.85	95.36	13.80	-12.25
280	93.80	97.66	14.43	-12.91
290	96.71	101.0	15.39	-13.91
298.15	99.13	103.7	16.18	-14.74
300	99.68	104.3	16.37	-14.93
310	102.7	107.6	17.38	-15.99
320	105.9	111.0	18.42	-17.08
330	109.1	114.3	19.50	-18.21
340	112.4	117.6	20.60	-19.37
350	115.8	120.9	21.75	-20.56
360	119.3	124.2	22.92	-21.79
370	123.0	127.5	24.13	-23.05

T / K	C^{o}_{pm} / J·K⁻¹·mol⁻¹	S^{o}_{m} / J·K⁻¹·mol⁻¹	$\Delta_0^T H^{o}_{m}$ / kJ·mol⁻¹	$\Delta_0^T G^{o}_{m}$ / kJ·mol⁻¹
380	126.7	130.8	25.38	-24.34
390	130.6	134.2	26.67	-25.66
400	134.7	137.5	27.99	-27.02
410	138.8	140.9	29.36	-28.41
420	143.2	144.3	30.77	-29.84
430	147.8	147.7	32.23	-31.30
440	152.7	151.2	33.73	-32.79
450	157.8	154.7	35.28	-34.32

[a] Form α of glycine corresponds to crystal structure deposited in the Cambridge Structural Database with refcode GLYCIN02 (see Table 5 in the main article).

[b] The combined expanded uncertainty of heat capacity $U_c(C_{pm})$ as well as of all calculated thermodynamic values (with 0.95 level of confidence, k=2) is: $U_c(X)$=0.1 X below 7 K; $U_c(X)$=0.03 X in temperature range (7 to 20) K; $U_c(X)$=0.005 X in temperature range (20 to 300) K; $U_c(X)$=0.01 X in temperature range (300 to 350) K; $U_c(X)$=0.02 X in temperature range (350 to 450) K, where X represents the heat capacity or the thermodynamic property. Values are reported with one digit more than is justified by the experimental uncertainty to avoid round-off errors in calculations based on these results.

[c] Extrapolated values.

Table S5. Standard thermodynamic functions of β-glycine[a] at p = 0.1 MPa.[b]

T / K	C^{o}_{pm} / J·K⁻¹·mol⁻¹	S^{o}_{m} / J·K⁻¹·mol⁻¹	$\Delta_0^T H^{o}_{m}$ / kJ·mol⁻¹	$\Delta_0^T G^{o}_{m}$ / kJ·mol⁻¹
1[c]	1.359×10^{-3}	5.105×10^{-4}	3.736×10^{-7}	-1.369×10^{-7}
2[c]	7.111×10^{-3}	2.966×10^{-3}	4.242×10^{-6}	-1.691×10^{-6}
3[c]	1.667×10^{-2}	7.546×10^{-3}	1.586×10^{-5}	-6.781×10^{-6}
4[c]	2.932×10^{-2}	1.400×10^{-2}	3.859×10^{-5}	-1.741×10^{-5}
5[c]	4.559×10^{-2}	2.221×10^{-2}	7.568×10^{-5}	-3.536×10^{-5}
6	6.730×10^{-2}	3.234×10^{-2}	1.316×10^{-4}	-6.246×10^{-5}
7	9.663×10^{-2}	4.481×10^{-2}	2.128×10^{-4}	-1.008×10^{-4}
8	1.362×10^{-1}	6.016×10^{-2}	3.283×10^{-4}	-1.530×10^{-4}
9	1.895×10^{-1}	7.913×10^{-2}	4.898×10^{-4}	-2.224×10^{-4}
10	2.603×10^{-1}	1.026×10^{-1}	7.130×10^{-4}	-3.128×10^{-4}
11	3.529×10^{-1}	1.315×10^{-1}	1.018×10^{-3}	-4.294×10^{-4}
12	4.713×10^{-1}	1.671×10^{-1}	1.427×10^{-3}	-5.781×10^{-4}
13	6.178×10^{-1}	2.105×10^{-1}	1.970×10^{-3}	-7.662×10^{-4}
14	7.919×10^{-1}	2.624×10^{-1}	2.672×10^{-3}	-1.002×10^{-3}
15	9.940×10^{-1}	3.238×10^{-1}	3.563×10^{-3}	-1.294×10^{-3}
16	1.225	3.952×10^{-1}	4.670×10^{-3}	-1.653×10^{-3}
17	1.484	4.770×10^{-1}	6.022×10^{-3}	-2.088×10^{-3}
18	1.772	5.699×10^{-1}	7.647×10^{-3}	-2.611×10^{-3}
19	2.086	6.740×10^{-1}	9.574×10^{-3}	-3.232×10^{-3}

T / K	C^{o}_{pm} / J·K⁻¹·mol⁻¹	S^{o}_{m} / J·K⁻¹·mol⁻¹	$\Delta_0^T H^{o}_{m}$ / kJ·mol⁻¹	$\Delta_0^T G^{o}_{m}$ / kJ·mol⁻¹
20	2.428	7.896×10^{-1}	1.183×10^{-2}	-3.962×10^{-3}
25	4.492	1.542	2.889×10^{-2}	-9.671×10^{-3}
30	7.078	2.583	5.763×10^{-2}	-1.987×10^{-2}
35	10.02	3.892	1.003×10^{-1}	-3.595×10^{-2}
40	13.13	5.431	1.581×10^{-1}	-5.917×10^{-2}
45	16.26	7.158	2.316×10^{-1}	-9.058×10^{-2}
50	19.34	9.031	3.206×10^{-1}	-1.310×10^{-1}
55	22.35	11.02	4.248×10^{-1}	-1.811×10^{-1}
60	25.25	13.09	5.439×10^{-1}	-2.413×10^{-1}
65	28.02	15.22	6.771×10^{-1}	-3.120×10^{-1}
70	30.63	17.39	8.238×10^{-1}	-3.935×10^{-1}
75	33.10	19.59	9.832×10^{-1}	-4.860×10^{-1}
80	35.42	21.80	1.155	-5.895×10^{-1}
85	37.60	24.01	1.337	-7.040×10^{-1}
90	39.67	26.22	1.530	-8.296×10^{-1}
95	41.63	28.42	1.734	-9.662×10^{-1}
100	43.51	30.60	1.947	-1.114
110	47.07	34.92	2.400	-1.441
120	50.41	39.16	2.887	-1.812
130	53.56	43.32	3.407	-2.224
140	56.55	47.40	3.958	-2.678
150	59.42	51.40	4.538	-3.172
160	62.18	55.32	5.146	-3.706
170	64.87	59.17	5.781	-4.278
180	67.49	62.95	6.443	-4.889
190	70.06	66.67	7.131	-5.537
200	72.61	70.33	7.844	-6.222
210	75.17	73.93	8.583	-6.943
220	77.88	77.49	9.348	-7.701
230	80.92	81.02	10.14	-8.493
240	84.47	84.54	10.97	-9.321
250	88.72	88.07	11.83	-10.18
260	89.77	91.52	12.71	-11.08
270	91.19	94.92	13.62	-12.01
273.15	91.81	95.99	13.90	-12.31
280	93.42	98.29	14.54	-12.98
290	96.45	101.6	15.49	-13.98
298.15[c]	99.52	104.3	16.29	-14.82

[a] Form β of glycine corresponds to crystal structure deposited in the Cambridge Structural Database with refcode GLYCIN (see Table 5 in the main article).

[b] The combined expanded uncertainty of heat capacity $U_c(C_{pm})$ as well as of all calculated thermodynamic values (with 0.95 level of confidence, k=2) is: $U_c(X)$=0.03 X below 20 K; $U_c(X)$=0.005 X in temperature range (20 to250) K; $U_c(X)$=0.01 X in temperature range (250 to 300), where X represents the heat capacity or the thermodynamic property. Values are reported with one digit more than is justified by the experimental uncertainty to avoid round-off errors in calculations based on these results. A heat capacity anomaly appears near 252 K, which is attributed to a ferroelectric-paraelectric transition.

[c] Extrapolated values.

Table S6. Standard thermodynamic functions of γ-glycine[a] at p = 0.1 MPa.[b]

T / K	C^{o}_{pm} / J·K^{-1}·mol^{-1}	S^{o}_{m} / J·K^{-1}·mol^{-1}	$\Delta_0^T H^{o}_{m}$ / kJ·mol^{-1}	$\Delta_0^T G^{o}_{m}$ / kJ·mol^{-1}
1[c]	1.003×10^{-4}	3.217×10^{-5}	2.431×10^{-8}	-7.858×10^{-9}
2[c]	9.469×10^{-4}	2.910×10^{-4}	4.435×10^{-7}	-1.386×10^{-7}
3[c]	3.796×10^{-3}	1.118×10^{-3}	2.577×10^{-6}	-7.774×10^{-7}
4[c]	1.067×10^{-2}	3.025×10^{-3}	9.368×10^{-6}	-2.733×10^{-6}
5[c]	2.441×10^{-2}	6.725×10^{-3}	2.620×10^{-5}	-7.423×10^{-6}
6	4.829×10^{-2}	1.311×10^{-2}	6.157E-05	-1.708×10^{-5}
7	8.566×10^{-2}	2.316×10^{-2}	1.273×10^{-4}	-3.486×10^{-5}
8	1.395×10^{-1}	3.792×10^{-2}	2.384×10^{-4}	-6.497×10^{-5}
9	2.120×10^{-1}	5.834×10^{-2}	4.125×10^{-4}	-1.126×10^{-4}
10	3.047×10^{-1}	8.529×10^{-2}	6.691×10^{-4}	-1.838×10^{-4}
11	4.183×10^{-1}	1.195×10^{-1}	1.029×10^{-3}	-2.856×10^{-4}
12	5.537×10^{-1}	1.615×10^{-1}	1.513×10^{-3}	-4.254×10^{-4}
13	7.125×10^{-1}	2.120×10^{-1}	2.144×10^{-3}	-6.114×10^{-4}
14	8.971×10^{-1}	2.713×10^{-1}	2.947×10^{-3}	-8.523×10^{-4}
15	1.109	3.403×10^{-1}	3.947×10^{-3}	-1.157×10^{-3}
16	1.349	4.194×10^{-1}	5.174×10^{-3}	-1.536×10^{-3}
17	1.617	5.091×10^{-1}	6.655×10^{-3}	-2.000×10^{-3}
18	1.914	6.098×10^{-1}	8.418×10^{-3}	-2.558×10^{-3}
19	2.238	7.218×10^{-1}	1.049×10^{-2}	-3.223×10^{-3}
20	2.590	8.455×10^{-1}	1.290×10^{-2}	-4.006×10^{-3}
25	4.732	1.642	3.096×10^{-2}	-1.010×10^{-2}
30	7.403	2.734	6.111×10^{-2}	-2.092×10^{-2}
35	10.41	4.098	1.056×10^{-1}	-3.790×10^{-2}
40	13.55	5.693	1.654×10^{-1}	-6.229×10^{-2}
45	16.70	7.471	2.411×10^{-1}	-9.513×10^{-2}
50	19.77	9.390	3.323×10^{-1}	-1.372×10^{-1}
55	22.75	11.42	4.386×10^{-1}	-1.892×10^{-1}
60	25.61	13.52	5.596×10^{-1}	-2.515×10^{-1}
65	28.32	15.68	6.945×10^{-1}	-3.245×10^{-1}
70	30.88	17.87	8.425×10^{-1}	-4.083×10^{-1}
75	33.27	20.08	1.003	-5.032×10^{-1}

T / K	C^{o}_{pm} / J·K⁻¹·mol⁻¹	S^{o}_{m} / J·K⁻¹·mol⁻¹	$\Delta_0^T H^{o}_{m}$ / kJ·mol⁻¹	$\Delta_0^T G^{o}_{m}$ / kJ·mol⁻¹
80	35.52	22.30	1.175	-6.092×10^{-1}
85	37.64	24.52	1.358	-7.262×10^{-1}
90	39.63	26.73	1.551	-8.544×10^{-1}
95	41.52	28.92	1.754	-9.935×10^{-1}
100	43.32	31.10	1.966	-1.144
110	46.73	35.39	2.417	-1.476
120	49.93	39.59	2.900	-1.851
130	52.94	43.71	3.415	-2.268
140	55.80	47.74	3.958	-2.725
150	58.55	51.68	4.530	-3.222
160	61.20	55.55	5.129	-3.758
170	63.78	59.33	5.754	-4.333
180	66.31	63.05	6.405	-4.945
190	68.81	66.70	7.080	-5.594
200	71.29	70.30	7.781	-6.279
210	73.78	73.83	8.506	-6.999
220	76.27	77.32	9.256	-7.755
230	78.77	80.77	10.03	-8.546
240	81.27	84.17	10.83	-9.370
250	83.80	87.54	11.66	-10.23
260	86.33	90.88	12.51	-11.12
270	88.89	94.19	13.38	-12.05
273.15	89.69	95.22	13.67	-12.35
280	91.45	97.46	14.29	-13.01
290	94.04	100.7	15.21	-14.00
298.15	96.16	103.4	15.99	-14.83
300	96.64	104.0	16.17	-15.02
310	99.26	107.2	17.15	-16.08
320	101.9	110.4	18.15	-17.16
330	104.6	113.5	19.18	-18.28
340	107.2	116.7	20.24	-19.43
350	109.9	119.8	21.33	-20.62
360	112.6	123.0	22.44	-21.83
370	115.3	126.1	23.58	-23.08
380	118.0	129.2	24.75	-24.35
390	120.8	132.3	25.94	-25.66
400	123.5	135.4	27.16	-27.00
410	126.2	138.5	28.41	-28.37
420	129.0	141.6	29.69	-29.77
430	131.7	144.6	30.99	-31.20

[a] Form γ of glycine corresponds to crystal structure deposited in the Cambridge Structural Database with refcode GLYCIN01 (see Table 5 in the main article).

[b] The combined expanded uncertainty of heat capacity $U_c(C_{pm})$ as well as of all calculated thermodynamic values (with 0.95 level of confidence, k=2) is: $U_c(X)$=0.03 X below 20 K; $U_c(X)$=0.005 X in temperature range (20 to 300) K; $U_c(X)$=0.01 X in temperature range (300 to 350) K; $U_c(X)$=0.02 X in temperature range (350 to 430) K, where X represents the heat capacity or the thermodynamic property. Values are reported with one digit more than is justified by the experimental uncertainty to avoid round-off errors in calculations based on these results.

[c] Extrapolated values.
